# Ultra-processed foods, incident overweight and obesity, and longitudinal changes in weight and waist circumference: the Brazilian Longitudinal Study of Adult Health (ELSA-Brasil)

**DOI:** 10.1017/S1368980019002854

**Published:** 2020-04

**Authors:** Scheine Leite Canhada, Vivian Cristine Luft, Luana Giatti, Bruce Bartholow Duncan, Dora Chor, Maria de Jesus M da Fonseca, Sheila Maria Alvim Matos, Maria del Carmen Bisi Molina, Sandhi Maria Barreto, Renata Bertazzi Levy, Maria Inês Schmidt

**Affiliations:** 1 Postgraduate Program in Epidemiology, Universidade Federal do Rio Grande do Sul, Faculdade de Medicina – Campus Saúde, R. Ramiro Barcelos 2400, Porto Alegre, RS 90035-003, Brazil; 2 National Health Technology Assessment Institute, CNPq, Porto Alegre, RS, Brazil; 3 Postgraduate Program in Food, Nutrition and Health, Universidade Federal do Rio Grande do Sul, Porto Alegre, RS, Brazil; 4 Food and Nutrition Research Centre (CESAN) – Hospital de Clínicas de Porto Alegre, Universidade Federal do Rio Grande do Sul, Porto Alegre, RS, Brazil; 5 Postgraduate Program in Public Health and School of Medicine & Clinical Hospital, Universidade Federal de Minas Gerais, Belo Horizonte, MG, Brazil; 6 National School of Public Health, Fundação Oswaldo Cruz, Rio de Janeiro, RJ, Brazil; 7 Postgraduate Program in Collective Health, Instituto de Saúde Coletiva, Universidade Federal da Bahia, Salvador, BA, Brazil; 8 Postgraduate Program in Nutrition and Health, Universidade Federal do Espírito Santo, Vitória, ES, Brazil; 9 Department of Preventive Medicine, School of Medicine, Universidade de São Paulo, São Paulo, SP, Brazil

**Keywords:** Ultra-processed food, Obesity, Weight gain, Food handling

## Abstract

**Objective:**

To evaluate the association of ultra-processed food (UPF) consumption with gains in weight and waist circumference, and incident overweight/obesity, in the Brazilian Longitudinal Study of Adult Health (ELSA-Brasil) cohort.

**Design:**

We applied FFQ at baseline and categorized energy intake by degree of processing using the NOVA classification. Height, weight and waist circumference were measured at baseline and after a mean 3·8-year follow-up. We assessed associations, through Poisson regression with robust variance, of UPF consumption with large weight gain (1·68 kg/year) and large waist gain (2·42 cm/year), both being defined as ≥90th percentile in the cohort, and with incident overweight/obesity.

**Setting:**

Brazil.

**Participants:**

Civil servants of Brazilian public academic institutions in six cities (*n* 11 827), aged 35–74 years at baseline (2008–2010).

**Results:**

UPF provided a mean 24·6 (sd 9·6) % of ingested energy. After adjustment for smoking, physical activity, adiposity and other factors, fourth (>30·8 %) *v*. first (<17·8 %) quartile of UPF consumption was associated (relative risk (95 % CI)) with 27 and 33 % greater risk of large weight and waist gains (1·27 (1·07, 1·50) and 1·33 (1·12, 1·58)), respectively. Similarly, those in the fourth consumption quartile presented 20 % greater risk (1·20 (1·03, 1·40)) of incident overweight/obesity and 2 % greater risk (1·02; (0·85, 1·21)) of incident obesity. Approximately 15 % of cases of large weight and waist gains and of incident overweight/obesity could be attributed to consumption of >17·8 % of energy as UPF.

**Conclusions:**

Greater UPF consumption predicts large gains in overall and central adiposity and may contribute to the inexorable rise in obesity seen worldwide.

The world has witnessed a progressive, major increase in the burden of obesity over recent decades. Since 1980, the prevalence has doubled in more than seventy out of 195 countries^(1)^ and obesity has become a major problem not only in high-income but also in low- and middle-income countries^(2)^. The Global Burden of Disease study estimates that, in 2015, obesity affected 603·7 million (95 % CI 588·2–619·8 million) adults and 107·7 million (95 % CI 98·7–118·4 million) children worldwide, leading to major morbidity and mortality^(1)^.

A better understanding of what has changed over recent decades that could possibly be associated with population weight gain (for example, forms of eating) is of paramount importance. Increased consumption of ultra-processed foods and beverages is a likely candidate^(3)^ as an increase in ultra-processed food intake has paralleled the obesity pandemic, replacing traditional local eating patterns throughout the world^(4–9)^.

A recent cross-sectional evaluation of nineteen European countries showed that a 1 percentage point greater consumption of ultra-processed foods was associated with a 0·25 percentage point greater obesity prevalence^(10)^. However, more direct evidence from longitudinal studies to document the role of ultra-processed foods in adiposity gain over time and in the incidence of overweight/obesity is scant. We found only one such study, based on university graduates in Spain^(11)^.

Our purpose was to investigate the prospective association of ultra-processed food and beverage consumption with gains in weight and waist circumference, as well as with incident overweight/obesity among those not having excess weight at baseline and with incident obesity among those overweight at baseline.

## Methods

### Study design and population

The Brazilian Longitudinal Study of Adult Health (ELSA-Brasil) is a multicentre cohort aiming primarily to address risk factors for and progression of diabetes, CVD and other related chronic diseases. As previously described^(12,13)^, between August 2008 and December 2010, we recruited 15 105 active and retired, non-pregnant employees aged 35–75 years from public institutions of higher education and research located in six Brazilian capital cities (Salvador, Belo Horizonte, Rio de Janeiro, São Paulo, Vitória and Porto Alegre) and applied a series of questionnaires as well as laboratory and clinical examinations^(14,15)^. Between 2012 and 2014, participants returned to the research centres for further examination, during which the follow-up weight was obtained. All participants provided written informed consent, and research protocols were approved by the ethics committee of all the institutions involved.

**Fig. 1:**
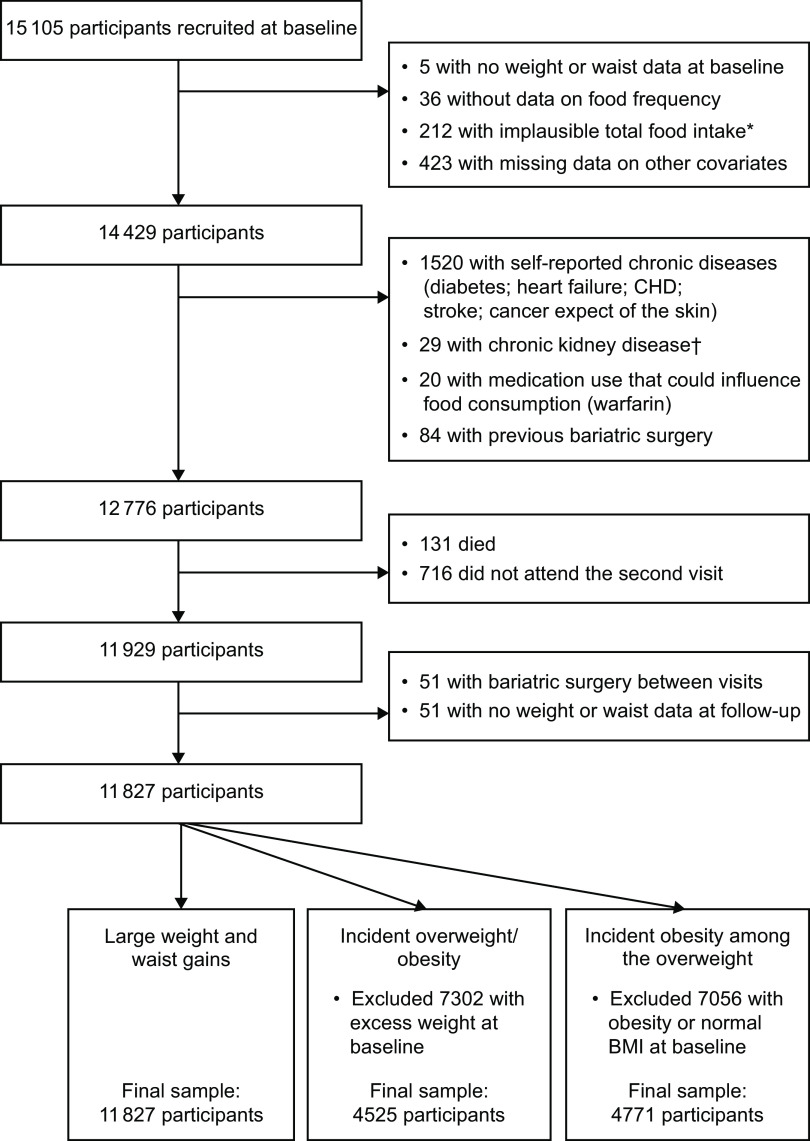
Flowchart of participants in the present study. *Implausible total food intake defined as <2510 or >25 104 kJ (<600 or >6000 kcal). †Chronic kidney disease defined as glomerular filtration rate of ≤45 ml/min per 1·73 m^2^

Among the 15 105 participants enrolled, we excluded participants without baseline weight or waist data (*n* 5), without data on food frequency (*n* 36), with an implausible total food intake (*n* 212), with missing data on other covariates at visit 1 (*n* 423) or with self-reported chronic diseases or medication use that could influence food consumption (*n* 1653). We also excluded 131 participants who died before visit 2 and an additional 716 did not attend visit 2. Finally, we excluded participants with bariatric surgery between visits (*n* 51) or with no weight or waist data at follow-up (*n* 51). The final sample consisted of 11 827 participants. For specific analyses related to incident overweight/obesity (BMI ≥ 25 kg/m^2^) and incident obesity (BMI ≥ 30 kg/m^2^), we made further specific exclusions related to baseline BMI, as shown in Fig. 1.

### Baseline measurements

We interviewed participants at baseline with standardized questionnaires to ascertain sociodemographic characteristics (age, sex, centre, race/skin colour, educational level, family income), previous medical history, smoking (current and previous) and physical activity, the latter defined using the International Physical Activity Questionnaire (IPAQ) section on leisure activity. The participant’s race/colour was self-reported. Per capita family income, also based on self-report, was calculated as the total family monthly income divided by the number of family members and expressed as a multiple of the Brazilian minimum wage.

We also obtained several anthropometric measures following internationally standardized protocols^(16,17)^. Waist circumference and weight were measured when fasting and with an empty bladder at the research clinics. During measurement, participants were dressed in standardized clothing without spectacles and other personal objects. We measured height to the nearest 0·1 cm (Seca model SE-216, Hamburg, Germany). We obtained waist circumferences with a 150 cm inelastic measuring tape (Mabis-Gulick, Waukegan, IL, USA) at the midpoint between the inferior edge of the costal border and the iliac crest in the mid-axillar line. We measured body weight with an electronic scale with maximum capacity of 300 kg (Toledo, São Bernardo do Campo, Brazil). Quality control measures were uniform across all centres. BMI was calculated as measured weight (in kilograms) divided by the square of measured height (in metres).

### Dietary assessment

Food consumption was evaluated at baseline through a previously validated FFQ with 114 food items^(18)^. For each item, we obtained the frequency of consumption in the last 12 months (with eight response options: ‘more than 3 times/day’, ‘2–3 times/day’, ‘once daily’, ‘5–6 times/week’, ‘2–4 times/week’, ‘once/week’, ‘1–3 times/month’ and ‘never/almost never’) and the number of portions consumed (using standardized portion sizes). The amount (grams per day) of each food item was calculated by multiplying the number of portions by the portion weight and the consumption frequency weight (3 for >3 times/d, 2 for 2–3 times/d, 1 for 1 time/d, 0·8 for 5–6 times/week, 0·4 for 2–4 times/week, 0·1 for 1 time/week, 0·07 for 1–3 times/month and 0 for never/almost never).

We employed the University of Minnesota Nutrition Data System for Research (NDSR) software to estimate the nutritional composition and energy value of recorded foods. For each of the food items, we imputed the respective 99th percentile consumption for participants with consumption above this percentile. Finally, we calculated the energy content of each food item by multiplying the daily food intake in grams by the energy in 100 g as estimated by the software (= intake grams × energy content per 100 g/100).

We applied the NOVA classification to allocate foods consumed into three groups according to the extent and purpose of their industrial processing: (i) non- or minimally processed foods and culinary ingredients; (ii) processed foods; and (iii) ultra-processed foods^(19)^. The energy consumption in each food group was then calculated by summing energy from the included food items, allowing the calculation of the relative contribution of each group to the total daily energy value.

### Outcomes

We calculated annual weight gain for each participant as the weight difference, in kilograms, between the baseline and follow-up visit, divided by the time, in years, between the two visits. We defined a large annual weight gain as equal to or greater than the 90th percentile (≥1·68 kg/year) gain in the sample. We defined a large annual gain in waist circumference similarly, considering its 90th percentile (≥2·42 cm/year). We investigated incident overweight and obesity (BMI ≥ 25 kg/m^2^) at follow-up among those not having excess weight (BMI < 25 kg/m^2^) at baseline and incident obesity (BMI ≥ 30 kg/m^2^) at follow-up among those overweight (25 kg/m^2^ < BMI ≤ 30 kg/m^2^) at baseline.

### Statistical analysis

We describe participant characteristics and outcomes using absolute and relative frequencies for categorical variables, and as mean and standard deviation or median and 25th–75th percentiles for continuous variables.

We characterized ultra-processed food consumption (percentage of total daily energy intake from these foods) as a continuous variable and expressed results for a 15 % increase in total energy consumed (approximately the interquartile range). We also categorized such consumption into quartiles based on the overall analytic sample and used the first quartile as a reference.

We analysed associations of ultra-processed food intake with a large annual weight or waist circumference gain and with incident overweight/obesity using Poisson regression with robust variance, progressively adjusting for age, sex, colour/race, centre, school achievement, and then smoking and physical activity. Finally, for weight/waist gain and for incident overweight/obesity, we additionally adjusted for baseline BMI, and for waist gain, we additionally adjusted for baseline waist circumference. In this full model, we tested interactions by categories of sex (men and women), race/colour (whites and non-whites) and age (<60 years *v*. ≥60 years).

We assessed the linearity of the associations between ultra-processed food consumption and these outcomes using restricted cubic splines^(20)^. We performed additional analyses: (i) to further adjust for total energy intake; (ii) to further adjust for fruit and vegetable consumption; and (iii) to investigate gains associated with non-beverage ultra-processed foods in which we adjusted also for ultra-processed beverage intake. We calculated the adjusted population-attributable fraction directly from the Poisson regressions^(21,22)^. All analyses were conducted with the statistical software package SAS version 9.4, except for the adjusted population-attributable fractions which were calculated using the statistical software package Stata version 11.1.

## Results

Of the 11 827 individuals analysed, 6507 (55·0 %) were women, 6169 (52·2 %) self-declared as being white and 6388 (54·0 %) had completed college/university. At baseline, mean age was 51·3 (sd 8·7) years, mean BMI was 26·8 (sd 4·6) kg/m^2^ and mean waist circumference was 90·6 (sd 12·5) cm. Mean total energy consumption was 10 979 (sd 3908) kJ/d (2624 (sd 934) kcal/d); 12 267 (sd 4063) kJ/d (2932 (sd 971) kcal/d) for men and 9924 (sd 3431) kJ/d (2372 (sd 820) kcal/d) for women. Foods and beverages classified as ultra-processed foods accounted for a mean of 24·6 (sd 9·6) % of total daily energy consumption; *in natura*, minimally processed or culinary ingredients for 64·1 %; and processed foods for 11·3 %. Table 1 lists the main foods and beverages classified as ultra-processed foods. Bread, sweets/candies, sweetened sodas/juices and salty pastries/chips accounted for more than 50 % of the total energy consumption from ultra-processed foods; other frequent items (>5 %) included cakes, processed meat, pasta/pizzas, cookies/crackers and mayonnaise/margarine/cream cheese.

**Table 1:** Frequency of consumption of specific ultra-processed foods and beverages and their contribution to energy intake. Brazilian Longitudinal Study of Adult Health (ELSA-Brasil), 2008–2010 (*n* 11 827)

	Ultra-processed food consumption			
Food item	Consumption frequency (% of total ultra-processed foods)		Contribution to energy intake (% of total daily energy intake)	
	Mean	sd	Mean	sd
Bread	23·1	17·3	5·6	4·8
Sweets, candies	13·2	11·0	3·4	3·4
Sweetened sodas/juices	9·4	11·5	2·4	3·2
Salty pastries, chips	8·7	8·7	2·1	2·2
Cakes	8·5	9·5	2·2	2·7
Processed meat	8·2	8·4	1·9	1·9
Pasta and pizzas	7·6	7·4	1·9	1·9
Cookies, crackers	7·5	10·1	1·8	2·5
Mayonnaise, margarine, cream cheese	6·7	7·4	1·6	1·7
Yoghurt (with additives)	3·9	6·2	0·9	1·4
Cereal bars	2·0	5·0	0·5	1·3
Distilled alcoholic beverages	1·0	4·3	0·2	0·8
Soup	0·2	0·8	0·1	0·2

Table 2 describes the sample characteristics according to quartile of daily consumption of ultra-processed foods when expressed as a percentage of daily energy intake. Compared with those in lower quartiles of ultra-processed food consumption, those in the fourth quartile were more frequently younger, women, white, with higher income, more educated, never smokers, with higher energy intake, less consumption of fruits and vegetables and greater ingestion of sweetened beverages. Sample characteristics were similar when we considered only those eligible for the outcomes of incident overweight/obesity and obesity.

**Table 2:** Characteristics of the study sample according to quartile of ultra-processed food consumption. Brazilian Longitudinal Study of Adult Health (ELSA-Brasil), 2008–2010 (*n* 11 827)

Characteristic	Ultra-processed food consumption (% of total daily energy intake)									
	Quartile 1 (0–17·79 %) (*n* 2956)		Quartile 2 (17·79–23·91 %) (*n* 2957)		Quartile 3 (23·91–30·84 %) (*n* 2957)		Quartile 4 (30·84–73·84 %) (*n* 2957)		Total (*n* 11 827)	
	Mean, *n* or median	sd, % or P25–P75	Mean, *n* or median	sd, % or P25–P75	Mean, *n* or median	sd, % or P25–P75	Mean, *n* or median	sd, % or P25–P75	Mean, *n* or median	sd, % or P25–P75
Age (years), mean and sd	53·6	8·5	51·7	8·6	50·7	8·5	49·3	8·7	51·3	8·7
Sex, *n* and %										
Female	1350	46·7	1576	53·1	1716	57·4	1865	62·7	6507	55·0
Skin colour/race, *n* and %										
Black	628	21·7	504	17·0	440	14·7	341	11·5	1913	16·2
Brown	972	33·6	902	30·4	789	26·4	658	22·1	3321	28·1
White	1174	40·6	1453	48·9	1652	55·2	1890	63·5	6169	52·2
Asian	75	2·6	86	2·9	76	2·5	66	2·2	303	2·6
Indigenous	43	1·5	24	0·8	33	1·1	21	0·7	121	1·0
Per capita family income (minimum wages/month), median and P25–P75	5	3–8	5	3–9	6	4–10	6	4–9	5	3–9
School achievement, *n* and %										
Less than elementary	264	9·1	131	4·4	113	3·8	80	2·7	588	5·0
Elementary	250	8·6	211	7·1	142	4·7	136	4·6	739	6·2
Secondary	1126	38·9	1056	35·6	1009	33·7	921	30·9	4112	34·8
College/university	1252	43·3	1571	52·9	1726	57·7	1839	61·8	6388	54·0
Smoking, *n* and %										
Never	1586	54·8	1696	57·1	1779	59·5	1866	62·7	6927	58·6
Ex-smoker	902	31·2	886	29·8	857	28·7	764	25·7	3409	28·8
Current	404	14·0	387	13·0	354	11·8	346	11·6	1491	12·6
Physical activity at leisure time (MET × min/week), median and P25–P75	240	0–960	264	0–960	244	0–954	240	0–929	240	0–960
BMI (kg/m^2^), mean and sd	26·8	4·6	26·8	4·6	26·8	4·5	26·8	4·8	26·8	4·6
Waist circumference (cm), mean and sd	91·6	12·4	90·7	12·2	90·2	12·5	89·7	12·7	90·6	12·5
Total daily energy intake (kJ/d), mean and sd	10 803	3837	10 979	3895	11 025	3933	11 104	3958	10 979	3908
Total daily energy intake (kcal/d), mean and sd	2582	917	2624	931	2635	940	2654	946	2624	934
Fruits and vegetable intake (% of total daily energy), mean and sd	11·0	6·3	9·8	5·4	9·0	5·2	7·6	4·4	9·4	5·5
Sweetened beverages (% of total daily energy), mean and sd	1·0	1·6	1·9	2·4	2·8	3·2	3·9	4·2	2·4	3·2

P25, 25th percentile; P75, 75th percentile; MET, metabolic equivalent of task.

After a mean of 3·8 (sd 0·4) years of follow-up, mean weight gain was 0·3 (sd 1·2) kg/year and mean waist circumference gain was 0·7 (sd 1·5) cm/year. Among those not overweight at baseline, 972 (21·5 %) became overweight or obese; among those overweight, 748 (15·7 %) became obese.

As seen in Table 3, incidence of large weight and waist gains increased monotonically with increasing consumption of ultra-processed foods, although incident overweight and obesity did not show such uniformly graded increases over the quartiles.

**Table 3:** Frequency of large (≥90th percentile) gains in weight and waist circumference and the incidence of overweight and obesity, according to quartile of ultra-processed food consumption. Brazilian Longitudinal Study of Adult Health (ELSA-Brasil), 2008–2010 (*n* 11 827)

Ultra-processed food consumption (% of total daily energy intake)	Large weight gain (*n* 11 827)		Large waist circumference gain (*n* 11 827)		Incidence of overweight and obesity* (*n* 4527)		Incidence of obesity† (*n* 4771)	
	Cases	%	Cases	%	Cases	%	Cases	%
Quartile 1 (0–17·79 %)	211	7·1	205	6·9	199	17·6	164	13·9
Quartile 2 (17·79–23·91 %)	286	9·7	265	9·0	233	21·0	200	16·2
Quartile 3 (23·91–30·84 %)	318	10·7	319	10·8	272	24·2	181	15·2
Quartile 4 (30·84–73·84 %)	378	12·8	394	13·3	268	23·1	203	17·4
Total	1193	10·1	1183	10·0	972	21·5	748	15·7

* Among those with BMI < 25 kg/m^2^ at baseline.

† Among those with BMI between 25 and 30 kg/m^2^ at baseline.

**Fig. 2:**
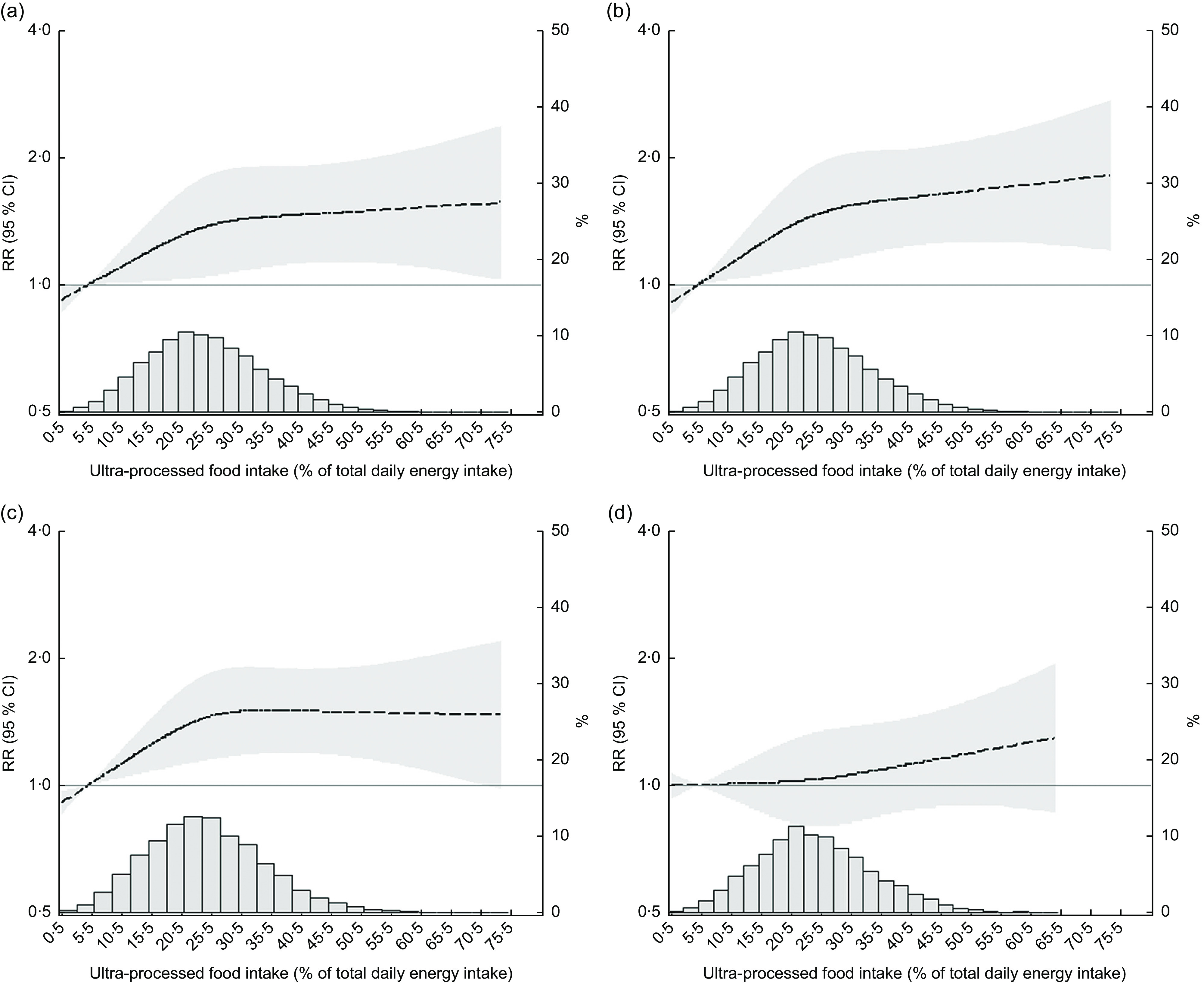
Associations (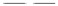, relative risk (RR); 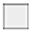, 95 % CI) of increasing intake of ultra-processed foods with (a) a major weight gain (≥90th percentile; ≥1·7 kg/year), (b) a major waist gain (≥90th percentile; ≥2·4 cm/year), (c) incident overweight or obesity among those without excess weight at baseline and (d) incident obesity among those overweight at baseline, after a mean 3·8-year follow-up. Brazilian Longitudinal Study of Adult Health (ELSA-Brasil), 2008–2010 (*n* 11 827). Associations were obtained through restricted cubic spline analyses adjusted for age, sex, colour/race, school achievement, per capita family income, smoking, physical activity and baseline waist (for waist gain) or BMI (for weight gain, incident overweight and obesity and incident obesity among those overweight). The *y*-axis to the right of each plot indicates the relative frequency (%) of the ultra-processed food intake displayed in the superimposed distribution curve

Restricted cubic spline regression, after adjusting for possible confounders, revealed statistically significant, positive relationships of greater ultra-processed food consumption with large weight and waist gains and with the incidence of overweight/obesity among those not overweight/obese at baseline, as seen by the 95 % confidence bands. The magnitude of these associations increased monotonically as ultra-processed food consumption expanded from low levels to approximately 25 % of total daily energy intake. Then, the associations with large weight and waist gains slowly plateaued (*P* for test of non-linearity = 0·23 and 0·17, respectively), while that with incident overweight/obesity completely flattened (*P* = 0·03 for non-linearity). In contrast, among those overweight at baseline, gradual increases in incident obesity were seen only with higher levels of ultra-processed food intake (approximately 20 % of total daily energy; *P* = 0·56 for non-linearity), although this increase in risk never achieved statistical significance (Fig. 2).

Table 4 shows the associations of ultra-processed foods with each outcome in progressively adjusted models. First, ultra-processed food intake was analysed continuously, with associations being expressed for a 15 % increase in total energy consumed as ultra-processed foods. Given the non-linear association for incident overweight/obesity with increased levels of ultra-processed food intake in restricted cubic spline analyses, we have not expressed this association with the intake analysed continuously. In crude analyses, the increment for each of the remaining outcomes was associated with moderately increased risk: large weight and waist gains (relative risk (RR) = 1·36, 95 % CI 1·26, 1·47; and RR = 1·42, 95 % CI 1·31, 1·53, respectively) and obesity (RR = 1·20, 95 % CI 1·09, 1·33). After adjustments, associations decreased but remained statistically significant for large weight and waist circumference gains: an increment of 15 % in consumption was associated with an increased risk of 12 and 15 % (RR = 1·12, 95 % CI 1·03, 1·22; and RR = 1·15, 95 % CI 1·06, 1·25), respectively. Consistent with the restricted cubic spline regression analyses (Fig. 2), associations for incident obesity among those overweight at baseline were not statistically significant (RR = 1·06, 95 % CI 0·96, 1·17).

**Table 4:** Association of ultra-processed food consumption (% of total daily energy intake) with large weight and waist circumference gains^*^, with incident overweight and obesity among those without excess weight at baseline and with incident obesity among those overweight at baseline, after a mean 3·8-year follow-up. Brazilian Longitudinal Study of Adult Health (ELSA-Brasil), 2008–2010 (*n* 11 827)

	Ultra-processed food consumption	Model 1		Model 2		Model 3		Model 4	
		RR	95 % CI	RR	95 % CI	RR	95 % CI	RR	95 % CI
Large weight gain (≥90th percentile: ≥1·68 kg/year) (*n* 11 827)	For each 15 % point increase	1·36	1·26, 1·47	1·13	1·04, 1·23	1·14	1·05, 1·24	1·12	1·03, 1·22
	Quartile 2	1·35	1·14, 1·61	1·17	0·98, 1·38	1·17	0·99, 1·39	1·15	0·97, 1·37
	Quartile 3	1·51	1·28, 1·78	1·20	1·01, 1·42	1·22	1·03, 1·44	1·20	1·02, 1·42
	Quartile 4	1·79	1·52, 2·10	1·28	1·08, 1·52	1·30	1·10, 1·54	1·27	1·07, 1·50
Large waist circumference gain (≥90th percentile: ≥2·42 cm/year) (*n* 11 827)	For each 15 % point increase	1·42	1·31, 1·53	1·14	1·05, 1·24	1·15	1·06, 1·25	1·15	1·06, 1·25
	Quartile 2	1·29	1·08, 1·54	1·11	0·93, 1·32	1·11	0·93, 1·33	1·11	0·94, 1·33
	Quartile 3	1·56	1·32, 1·84	1·21	1·02, 1·43	1·23	1·04, 1·46	1·23	1·04, 1·46
	Quartile 4	1·92	1·64, 2·26	1·30	1·10, 1·55	1·33	1·12, 1·57	1·33	1·12, 1·58
Incident overweight/obesity (BMI ≥ 25 kg/m^2^)* (*n* 4527)	For each 15 % point increase		(non-linear association in restricted cubic spline regression)						
	Quartile 2	1·19	1·00, 1·41	1·18	0·99, 1·40	1·17	0·99, 1·39	1·14	0·98, 1·33
	Quartile 3	1·37	1·16, 1·61	1·35	1·14, 1·60	1·34	1·13, 1·59	1·36	1·18, 1·57
	Quartile 4	1·31	1·11, 1·54	1·29	1·08, 1·54	1·29	1·08, 1·53	1·20	1·03, 1·40
Incident obesity among the overweight (BMI ≥ 30 kg/m^2^)† (*n* 4771)	For each 15 % point increase	1·20	1·09, 1·33	1·12	1·01, 1·25	1·13	1·01, 1·26	1·06	0·96, 1·17
	Quartile 2	1·17	0·97, 1·42	1·11	0·92, 1·34	1·12	0·92, 1·35	1·12	0·95, 1·32
	Quartile 3	1·10	0·90, 1·34	1·02	0·84, 1·24	1·03	0·85, 1·26	1·01	0·85, 1·21
	Quartile 4	1·25	1·04, 1·51	1·10	0·90, 1·34	1·11	0·91, 1·36	1·02	0·85, 1·21

RR, relative risk.

Model 1: crude.

Model 2: plus age, sex, colour/race, centre, income and school achievement.

Model 3: plus smoking and physical activity.

Model 4: for incident overweight/obesity and weight gain, plus baseline BMI; for waist gain, plus waist circumference at baseline.

Quartile 1 is always the reference quartile.

* Among those with BMI < 25 kg/m^2^ at baseline.

† Among those with BMI between 25 and 30 kg/m^2^ at baseline.

As also shown in Table 4, comparing the fourth with the first quartile of ultra-processed food consumption, we again found moderate increased risk for the four outcomes. In crude analyses associations were all statistically significant, with increased risk of 79, 92, 31 and 25 % for a large weight gain, a large waist gain, incident overweight/obesity and incident obesity (RR = 1·79, 95 % CI 1·52, 2·10; RR = 1·92, 95 % CI 1·64, 2·26; RR = 1·31, 95 % CI 1·11, 1·54; and RR = 1·25, 95 % CI 1·04, 1·51), respectively. After multiple adjustments, associations decreased somewhat and remained statistically significant for all outcomes except incident obesity among those overweight at baseline, with increased risk of 27, 33, 20 and 2 % for these same outcomes (RR = 1·27, 95 % CI 1·07, 1·50; RR = 1·33, 95 % CI 1·12, 1·58; RR = 1·20, 95 % CI 1·03, 1·40; and RR = 1·02, 95 % CI 0·85, 1·21), respectively. The only interaction that was statistically significant was that for race/colour groups (*P* = 0·02) when evaluating a large weight gain, with non-whites showing a stronger association.

In additional models (not shown in Table 4), associations remained essentially unchanged after further adjustment for total energy intake, having increased risk of 27, 36, 22 and 2 % for a large weight gain, a large waist gain, incident overweight/obesity and incident obesity (RR = 1·27, 95 % CI 1·07, 1·51; RR = 1·36, 95 % CI 1·14, 1·61; RR = 1·22, 95 % CI 1·04, 1·42; and RR = 1·02, 95 % CI 0·85, 1·21), respectively. Only minimal changes were seen also after further adjustment for fruit and vegetable consumption, the increased risks being 33, 38, 22 and 1 % for these same outcomes (RR = 1·33, 95 % CI 1·11, 1·58; RR = 1·38, 95 % CI 1·16, 1·64; RR = 1·22, 95 % CI 1·04, 1·42; and RR = 1·01, 95 % CI 0·85, 1·21), respectively.

We also investigated these associations after excluding sweetened beverages from the ultra-processed foods classification. In this analysis, we added sweetened beverage consumption to model 4 adjustments. Associations were similar, with increased risk of 34, 42, 24 and 3 % for a large weight gain, a large waist gain, incident overweight/obesity and incident obesity (RR = 1·34, 95 % CI 1·13, 1·58; RR = 1·42, 95 % CI 1·20, 1·69; RR = 1·24, 95 % CI 1·06, 1·44; and RR = 1·03, 95 % CI 0·87, 1·22), respectively.

Population-attributable fraction analyses showed that 14·1, 15·2 and 14·9 % of large weight gains, large waist gains and incident cases of overweight/obesity, respectively, could be attributable to high (greater than the first quartile) consumption of ultra-processed foods and beverages.

## Discussion

We found an approximately 20–30 % greater risk of large weight and waist circumference gains and greater incidence of overweight/obesity over 3·8 years of follow-up, comparing those in the highest with those in the lowest quartile of ultra-processed food consumption. Although these associations are of small to moderate size, given the high frequency of ultra-processed food consumption, adjusted population-attributable fractions for all outcomes were approximately 15 %, demonstrating the large potential public health significance of this exposure.

The role of processed foods in health and disease has been investigated in recent decades, more specifically with respect to sweetened beverages and processed meats, generally showing positive associations with weight gain and obesity^(23–25)^. Our use of the NOVA classification of processed foods allowed us to include other foods suffering similar processing mechanisms and thus to assess ultra-processing of foods more broadly. Application of this classification in Europe revealed linearly greater obesity rates according to the frequency of ultra-processed food consumption across countries^(10)^. Positive cross-sectional associations have been also observed in studies conducted in Brazil and other Latin American countries^(26–28)^.

To our knowledge, the only other prospective study reporting the association of ultra-processed foods with incident overweight/obesity is that of Mendonça *et al*.^(11)^. Investigating a cohort of university alumni in Spain, they found similar associations (hazard ratio = 1·26, 95 % CI 1·10, 1·45 for consumption of 6·1 (sd 0·9) *v*. 1·5 (sd 0·9) portions of ultra-processed foods daily). Our results significantly extend their findings by including adults residing in a middle-income country with a wider range of educational achievement, ethnicity and age, which is important considering the recent trends for increasing consumption of ultra-processed foods in these countries^(4)^. By investigating a wider scope of obesity/central obesity outcomes, we were able to demonstrate a similar association with gain in waist circumference. Moreover, after excluding sweetened beverages from the NOVA classification of ultra-processed foods, in a secondary analysis, we found similar associations, demonstrating that the risk associated with ultra-processed foods does not result solely from sweetened beverages.

The use of the NOVA classification has recently been criticized^(29)^ as lacking support from human studies demonstrating risk; being too dependent on added sugars; lacking underlying biological mechanisms to explain possible harm; and being too broad to be useful.

In fact, evidence to support its risk is growing, now extending also to incident cancer^(30)^ and overall mortality^(31)^. With regard to obesity, our findings confirm the role of ultra-processed foods in incident overweight and obesity found by Mendonça *et al*. and by one small study of weight gain in pregnancy^(32)^, as well cross-sectional evidence originated in different countries and settings, which almost uniformly suggests risk^(10,27,33–35)^. Moreover, our findings that associations remained after exclusion of sugar-sweetened beverages suggest that excess sugar^(36)^ is not the only culprit.

Possible mechanisms to explain the associations between ultra-processed foods and obesity can be outlined, the first one being the increased energy intake associated with the consumption of ultra-processed foods^(37)^. Ultra-processed food products, compared with the other groups of foods, show greater energy density, as well as greater total and saturated fat, *trans* fats and sugar, and less fibre, protein and potassium, which illustrates their generally poor nutritional value^(38)^. Being designed to favour consumption and satiate less^(39)^ they can be consumed more frequently and in larger portions, contributing to increased energy intake. Being relatively low in protein, they may also increase intake because of the proposed dominant drive for protein intake^(40)^. However, our findings and those of Mendonça *et al*. showing increased risk even after adjusting for daily total energy intake suggest that additional mechanisms are at play. It is also possible that the intake of ultra-processed foods dislocates more healthy foods from the daily diet. However, our associations changed only minimally when we adjusted for fruits and vegetables, indicating that associations do not result solely from eating less of these foods.

Another line of potential mechanism involves the food additives in ultra-processed foods. Emulsifiers, common in ultra-processed products, led to disruption in the intestinal mucus barrier in mice, producing chronic inflammation and the metabolic syndrome^(41)^, a phenotype linked to weight gain. Eating a Western (*v*. more traditional) diet, which increases consumption of food additives, produces alteration in the distinct combination of bacteria in the intestine, the result of which may be a more dysfunctional metabolic status^(42,43)^. Consumption of soda, which includes multiple additives, has been associated with a lower level of *Akkermansia muciniphila*, believed to be protective against obesity and type 2 diabetes. Snack and junk food products, often characterized by the long list of additives among their ingredients, have been associated with higher counts of *Escherichia coli* and a lesser presence of lactobacilli and butyrate-producing *Firmicutes* species, these alterations believed to lead to detrimental inflammatory effects within the gut milieu^(44)^.

Finally, although the concept of ultra-processed food may be broad, it can be useful for public health, as shown by its application as the basis of current national nutritional guidelines in Brazil and Uruguay^(45,46)^.

The consumption of ultra-processed foods has increased remarkably in the last decades worldwide, replacing the consumption of minimally processed and fresh foods. In Canada, the dietary share of ultra-processed products in the average household food basket in the 1930s was 24·4 % and in the early 2000s, 54 %^(6)^. Other high-income countries also show major contributions of ultra-processed foods to energy intake, being larger when compared with that of middle-income countries^(6,47)^. This larger share of consumption may result from marketing strategies similar to those used for selling tobacco products^(48)^. In middle-income countries consumption of ultra-processed foods has increased more recently and more rapidly^(4)^. In Brazil, ultra-processed foods accounted for 19·2 % of total energy intake in 1987–1988^(3)^, reaching 25·4 % in 2008–2009^(5)^, a percentage which was also found in the baseline of the ELSA-Brasil population^(49)^. Of note also, we found greater consumption among the more educated/higher-income individuals, consistent with previous findings in Brazil^(5)^. The rising trend in ultra-processed food consumption in low- and middle-income countries is likely to continue and may change from its current sociodemographic distribution. In Brazil, 61 % of food advertising on open television was for ultra-processed foods *v*. only 7 % for *in natura* or minimally processed foods^(50)^. These factors, all together, influence food choice and eating behaviours, and thus alter eating patterns in the population, frequently without individual awareness or control^(51)^.

Our study has some limitations. First, FFQ, although traditionally used to assess nutritional intake in epidemiological studies, are imprecise and may under- or overestimate total energy intake. However, our measure of consumption of ultra-processed foods as a percentage of total energy intake minimizes problems related to under- or overestimation. Our version was not specifically designed for use with the NOVA classification. Despite this, the frequency of ultra-processed food consumption we report is consistent with that of a nationwide representative survey^(38)^. We cannot rule out reporting bias, although the hypothesis that ultra-processed foods cause weight gain was not recognized in Brazil during baseline interviews. Moreover, non-differential misinformation, if present, is more likely to bias towards the null. Second, our follow-up of approximately 4 years resulted in our outcome being assessed over a relatively short period of time. Third, although adjustments were made for possible confounders in statistical analyses, it is not possible to rule out residual confounding.

Our study also has strengths. First, this is a large contemporary cohort study with small losses to follow-up (<6 %). Second, we performed highly standardized measurements, including waist circumference, with strict quality control. Third, our spline analyses permit a detailed evaluation of change in risk across the spectrum of ultra-processed food consumption. Fourth, our various sensitivity analyses confer robustness to the interpretation of our findings.

Our findings are consistent with previous studies^(10,11)^, supporting the contention that high consumption of ultra-processed foods may contribute to the current obesity epidemic in Brazil. Although issues of external validity need to be taken into consideration, our finding may apply also to the obesity epidemic in other settings as many have a similar pattern of increasing consumption of ultra-processed foods over the period of rising obesity^(10)^. Consumption of ultra-processed foods has increased globally as marketing strategies have progressively targeted an increasing fraction of the world’s population, initially in high-income countries and more recently in low- and middle-income countries^(4)^.

Public policies to reduce ultra-processed foods are now being implemented in different countries. Policies restricting or banning the sale of sugar-sweetened beverages and confectioneries in schools and other institutional or commercial settings have been implemented in several countries^(52)^. Brazil and Uruguay have developed dietary guidelines explicitly referring to the category of ultra-processed foods and advising the population not to replace minimally processed foods and their culinary preparations with ultra-processed food and drink products^(45,46)^.

## Conclusion

In conclusion, we provide prospective evidence that consumption of ultra-processed foods and beverages is related to gains in overall and central adiposity, and to incident overweight/obesity among those not so at baseline, in a broad, free-living population. Public policies aimed at reducing the consumption of ultra-processed foods may help revert the to-date inexorable rise in obesity in Brazil and elsewhere.
